# A latent profile analysis of aggression and prosocial behavior in relation to adolescent wellbeing

**DOI:** 10.3389/fpsyg.2025.1545055

**Published:** 2025-07-11

**Authors:** Lénia Carvalhais, Paula Vagos

**Affiliations:** ^1^Departament of Psychology and Education, University Portucalense, Porto, Portugal; ^2^Life Quality Research Centre (CIEQV), Santarém, Portugal; ^3^William James Center for Research, University of Aveiro, Aveiro, Portugal

**Keywords:** aggressive behaviors, prosocial behaviors, subjective wellbeing, adolescents, latent profile analysis

## Abstract

**Introduction:**

Aggressive and prosocial behaviors have often been addressed as opposing constructs, namely in their opposite association with subjective wellbeing. Alternatively, the Resource Control Theory assumes that individuals may resort to both aggressive and prosocial behaviors as strategic ways to obtain individual and social resources, which are particularly relevant in adolescence. This bistrategic use of social behaviors may be particularly noticeable when considering the overt and indirect forms of aggression but these forms have not been considered before in relation to prosociality.

**Method:**

The current work explored profiles based on prosocial and aggressive behavior (i.e., relational, reputational and overt) and compared those profiles on different dimensions of subjective wellbeing. Participants were 350 students aged 11 to 18 years old (Mage = 13.40) attending the 7th through 9th school grades, of which 191 (54.6%) were female. They reported on the practice of overt aggression, relational aggression, reputational aggression, and prosocial behavior and their emotional, social, and psychological wellbeing.

**Results:**

Using latent profile analyses, typical and bistrategic profiles were found. Mean comparisons further showed that participants in these two profiles differed in all forms of aggressive behavior but not in the practice of prosocial behavior nor emotional, social, and psychological wellbeing, which might have been driven by prosociality.

**Discussion:**

When considering the forms of aggression in a community and age-diverse sample, adolescents seem resourceful in responding to their inter and intrapersonal developmental needs while maintaining their wellbeing. Promoting prosocial behavior as a valid alternative to aggression may have to be rooted in the intention with which these acts are practiced so that both are openly seen as ways of sustaining not only the others' but also one's own welfare.

## 1 Introduction

Social behaviors are crucial during adolescence because they allow adolescents to take advantage of socialization opportunities relevant to general human development as well as to subjective wellbeing (Hirani et al., 2022; Sharma and Tomer, 2018). Such behaviors may relate to adaptive or non-adaptive social functioning and wellbeing, including prosocial and aggressive behaviors, which have been considered as opposite or negatively correlated constructs (e.g., Dobbelaar et al., 2021; Padilla-Walker et al., 2018). Instead, Hawley's Resource Control Theory (RCT; Hawley, 1999) proposes that antisocial behaviors (e.g., aggression) and cooperative strategies (e.g., prosocial) are not necessarily mutually exclusive, but can co-exist and be practiced as a bistrategic way of obtaining gains, be those gains material or social status and dominance. Within the RCT framework, both prosocial and coercive resource control strategies may stem from the same underlying intention to acquire personal and social gains, and the individual may preferably opt for prosocial strategies, coercive strategies or both. Individuals who strategically resort to both strategies based on their judgment of the contextual demands of a given social event may be the ones obtaining the highest gains (Hawley, 2006). The social benefits of being bistrategic may be particularly enhanced if one practices the indirect forms of aggression (i.e., reputational and relational aggression), which are focused on gaining social allies or status at the expense of the victims' loss (Young et al., 2006).

Integrating the forms of aggression within the RCT framework may be useful to better identify profiles of adolescents' social behavior and, consequently, conceptualize and intervene to promote beneficial social behaviors. Though the motives for relating with peers may change throughout adolescence (namely from sharing interests and attitudes, to providing a context for the experimentation of one's identity and to becoming intimate and supporting allies), peers play an important role in the adolescents' development and wellbeing (Salmela-Aro, 2011). This may make adolescents particularly prone to use whatever strategy is available, including prosocial and both relational and overt aggression. The current work is based on the RCT premises, and its primary goal is to explore social behavior profiles found in a community adolescent sample, to ascertain if aggressive and prosocial behavior exist as separate and/or cooccurring behaviors, particularly considering the forms of aggression being practiced. Furthermore, this works' secondary goal is to validate the content of those social behaviors profiles based on sex distribution of participants and on self-reported social, emotional and psychological wellbeing.

### 1.1 Prosocial, aggressive and bistrategic behaviors

Prosocial behaviors are one of the most critical social behaviors that develop throughout adolescence and lead to the achievement of collective goals and the development of reciprocal relationships and social belonging (Crone and Achterberg, 2022). This behavior refers to acts that are practiced with the intention and consequence of benefiting others (e.g., helping, sharing, donating, complementing, supporting, empathizing, volunteering), that are valued by one's cultural or societal group (Pfattcheicher et al., 2022; Sebastian et al., 2010). Instead, aggressive behaviors are practiced toward a victim with the intention to cause physical or psychological damage to them (Bettencourt et al., 2017). Those acts can be perpetrated in different forms, namely through overt forms (Prinstein et al., 2001) or indirect forms such as relational (Crick and Grotpeter, 1995; Prinstein and Cillessen, 2003; Voulgaridou and Kokkinos, 2023) and reputational (Xie et al., 2002) aggression. Overt forms of aggression include hitting, teasing, or kicking. Relational aggression uses relationships as weapons to harm the victim (e.g., excluding someone from social activities) by manipulating between-peer relationships (De Los Reyes and Prinstein, 2004). Reputational aggression, in turn, is a way of damaging another person's social reputation by using others to cause damage to that person's status within the group hierarchy (e.g., telling others to dislike someone or spreading gossip or rumors; Malamut et al., 2018; Xie et al., 2002).

Though seemingly opposite constructs, RCT (Hawley, 1999) proposes that prosocial and aggressive behaviors may co-exist and be practiced by the same individual as a way of responding to diverse needs arising within different social interactions where the individual intends to be successful in social competition. Youth who use both behaviors (in other words, who adopt bistrategic behavior) may actually be displaying increased social competence by being able to resort to a wider social behavior repertoire to achieve and/or gain social status. Based on this assumptions, Hawley (2003) proposed to categorize children based on their self-reported use of coercive and/or prosocial strategies into five groups: aggressors/coercive controllers who practice high aggressive and low prosocial behaviors, non-controllers who practice low aggressive and low prosocial behaviors, typical who practice within average aggressive and prosocial behaviors, prosocial who practice low aggressive and high prosocial behaviors, and bistrategic who practice high aggressive and high prosocial behaviors. The bistrategic group emerged as being aggressive but also liked by their peers, socially skilled and adjusted (Hawley, 2003). As applied to adolescents, the bistrategic group was further found to perceive their friendships as fun and intimate, though also conflicting (Hawley, 2003) and to be nominated as having prominence and access to resources within the group, belonging to high status groups, and perceiving their own popularity as high (Wurster and Xie, 2014). Moreover, using a momentary approach (i.e., daily) to the social behaviors of adolescents, Arbel et al. (2022) found that prosocial and aggressive behavior co-exist in the day-to-day of adolescents and that such diversity is needed to navigate the complexities of the adolescents' daily peer interactions and school life.

Still, these groups have not been apparent in other previous works that used person-centered approaches to the data and provided evidence for diverse (and sometimes inconsistent) profiles of the combination of prosocial and aggressive behaviors. Using peer nomination, Berger et al. (2015) explored profiles and found evidence for typical, prosocial and aggressive groups and McDonald et al. (2015) encountered non-controllers, aggressive, prosocial and bistrategic groups; both works used mid-adolescent samples. Though both works considered nominations about overt and relational aggression, these nominations were combined to be used as a single aggression measure, meaning that the diverse impact of these forms of aggression was not explored. Nantel-Vivier et al. (2014) and Ettekal and Mohammadi (2020) addressed developmental trajectories in longitudinal works and found a trajectory portraying the continued co-occurrence of at least moderate levels of both prosocial and aggressive behavior, in addition to mainly aggressive (Nantel-Vivier et al., 2014; Ettekal and Mohammadi, 2020) and to mainly prosocial trajectories (Ettekal and Mohammadi, 2020). None of these works considered how diverse profiles may be dependent on diverse forms of aggression. Instead, Hartl et al. (2019) considered the contribution of both overt and relational aggression and used a person-centered approach to explore social profiles. There was evidence for popularity being based on using strategic, aggressive, prosocial and typical social strategies; the groups differed in their practice of both relational and overt aggression, in addition to prosocial behaviors.

### 1.2 Prosocial, aggressive and bistrategic behaviors in relation to sex and wellbeing

Sex-distribution and mean values differences across groups have been explored before and may serve to validate the constructs portrayed by social behaviors profiles. Previous works found that females report more practice of prosocial behavior than males (Queirós and Vagos, 2016; Stubbs-Richardson et al., 2018). Instead, though not consensual (see, for example, Card et al., 2008), previous works have often found male adolescents to report more practice of all forms of aggression (Queirós and Vagos, 2016; Vagos and Carvalhais, 2020), including relational aggression; this finding seems to be specifically related to European samples (Voulgaridou and Kokkinos, 2023). Accordingly, and considering the social profiles anticipated by RCT, previous evidence shows that male adolescents are more prevalent in the aggressive group, that female adolescents are more prevalent in the prosocial group, and that male and female adolescents are equally distributed in the bistrategic group (Hartl et al., 2019; McDonald et al., 2015).

Previous work has also considered these groups in relation to adaptive outcomes but has mostly focused on interpersonal constructs, and found that bistrategic children and adolescents have similar outcomes to prosocial ones, namely concerning peer acceptance (Ettekal and Mohammadi, 2020; Hartl et al., 2019), high popularity (Hartl et al., 2019; Reijntjes et al., 2018), and individual and social status within the peer group (Wurster and Xie, 2014), even if being and aggressor and victim is also a feature of being bistrategic (Hawley, 2003; Reijntjes et al., 2018). Instead, other works have focused on individual motives to pursue either aggressive, prosocial or bistrategic social behaviors, namely based on internalized norms on how popularity may be achieved (Laninga-Wijnen et al., 2020) and about what the individual wants to obtain from interacting with others (McDonald et al., 2015). As such, intrapersonal features experienced by these diverse groups have seldom been considered, even if prosocial and aggressive behaviors have individually been associated with such types of outcomes.

Subjective perception of wellbeing may be a relevant intrapersonal variable to consider within this framework and add to previous findings about interpersonal gains associated with diverse social behavior profiles. The Mental Health Continuum Model, which is based on positive psychology, suggests that subjective wellbeing is a complex and multidimensional concept encompassing emotional, psychological, and social dimensions (Keyes, 2002). Emotional wellbeing is related to positive emotions, including the individuals' perceptions of happiness, interests, and balanced positive and negative feelings. The psychological and social dimensions of wellbeing are assessed through positive functioning, individual accomplishments, and life satisfaction. The psychological dimension implies positive self-assessment and acceptance within a continuous personal development process toward the individual's goals and life projects. As for the social dimension of wellbeing, it refers to the individuals' perception of society as meaningful to them, in as much as it enables personal development, and the individual considers themselves accepted and integrated into their social context (Iasiello et al., 2022; Kennes et al., 2020). Though conceptualization of wellbeing has been widely validated across diverse cultures and sample (Iasiello et al., 2022), wellbeing has not often been considered in relation to the adaptive or maladaptive indicators associated with prosocial or aggressive behavior.

Prosocial behavior has been associated with a plethora of positive mental health outcomes, including increased positive affect, family and community impact, social network, overall mental health, and satisfaction with life, as reviewed by Curry et al. (2018). Specifically with adolescent samples, previous works found that overall prosocial behavior was associated with increased life satisfaction over time (Son and Padilla-Walker, 2019) and was linked to higher optimism, lower depressive symptoms, and better academic achievement, possibly by allowing adolescents to be accepted by peers within the classroom (Oberle et al., 2023). Alternatively, the diverse forms of aggression have been found to associate differently with psychosocial adjustment (including the practice of prosocial behavior; Card et al., 2008) and may also be considered differently in relation to obtaining social resources. Specifically, indirect forms of aggression may be particularly advantageous for increasing one's social status (Dyches and Mayeux, 2015) and social dominance (Ingram, 2014). Practicing aggression has been associated with various negative mental health outcomes, including diminished subjective wellbeing, greater emotional and behavioral problems (Arslan et al., 2021), and diminished psychological health as referring to self-esteem and purpose in life (Stein et al., 2007), as well as low life satisfaction (Estévez et al., 2009).

### 1.3 Present study

RCT proposes that individuals may resort to diverse social strategies to obtain social gains, and previous empirical findings have validated that adolescents may be grouped via peer nomination into different profiles based on their use of those strategies to achieve popularity (e.g., Hartl et al., 2019). Still, it has not been considered if those strategies may combine in relation to the self-reported practice of aggressive and prosocial behavior, while also considering the diverse forms of aggression that may elicit diverse social outcomes. So, the current study's primary goal is to explore profiles of adolescents' social behaviors based on self-reports of overt, relational, and reputational aggression and prosocial behavior, specifically providing help or being present to others in need. Based on the RCT, at least five profiles could be uncovered: aggressors, non-controllers, typical, prosocial, and bistrategic. Still, previous empirical works have not found those five groups. In fact, the non-controller group has not become apparent, and results have been inconsistent regarding the other groups. So, the current work will adopt an exploratory and person-centered approach to data analysis. Specifically, a latent profile analysis (LPA) approach to data analyses will be used that allows uncovering the best-fitting latent groups based on estimates probability of empirical data. LPA allows individuals to be categorized based on their patterns of responses to a combined and predefined group, and that categorization determines both the number of profiles that optimally represent the data and their size (Nylund et al., 2007). We hypothesize that our findings will replicate those of previous empirical work using a similar approach to data analyses (i.e., Hartl et al., 2019) and four profiles will be a good fit for the data, namely typical, aggressor, prosocial and bistrategic.

To validate the content of these profiles, an analyses of gender distribution within profiles will be conducted. The hypothesis is that male adolescents will be more prevalent in the mainly aggressor group (e.g., Hartl et al., 2019; Hawley, 2003; Queirós and Vagos, 2016), and female adolescents will be more prevalent in the mainly prosocial group (e.g., Hartl et al., 2019; Hawley, 2003; Queirós and Vagos, 2016; Wurster and Xie, 2014), with a similar prevalence of males and females in the bistrategic group (Hartl et al., 2019; Hawley, 2003). Social behavior profiles will also be compared based on self-reported subjective wellbeing, including its three dimensions of emotional, psychological, and social wellbeing. The hypotheses are that aggressive adolescents report lower subjective wellbeing and that prosocial adolescents report higher subjective wellbeing. As for the bistrategic group, it is hypothesized that they will also report increased subjective wellbeing, assuming that prosociality compensates for aggression, as it has been found to compensate in relation to peer acceptance (Ettekal and Mohammadi, 2020).

## 2 Method

This is a cross-sectional study that collected all data at a single point in time to observe and describe a given phenomenon as it occurs. This methodology aligns with this works' goals of primarily identify latent profiles based on social behaviors and secondarily describe those profiles based on sex and wellbeing. Likewise, the assumption of the existence of diverse resource control groups based on RCT has often been tested using cross-sectional designs (e.g., Berger et al., 2015; Nantel-Vivier et al., 2014).

The minimum sample size was defined *a priori* based on the assumption that a maximum of five groups would be found concerning this study's primary goal and would be compared within this study's secondary goal. Based on the G^*^Power *software* and anticipating a one-way ANOVA between five groups with an expected effect size of 0.25, an error probability of 0.05 and power set at 0.95, a minimum total sample size was set at 305 participants.

### 2.1 Participants

This work received approval from the Ethics Committee of the Department of Psychology and Education of the University of Coimbra. Then, three schools conveniently selected for being located in the northern Portugal region were contacted to participate in this research, providing access to their students. The inclusion criteria were students attending the 7th through 9th grades; the exclusion criterion was students who had a specific learning disability that might impair their ability to understand the items of the self-report protocol autonomously. The schools sent and collected informed consent forms from parents and legal guardians of students attending the 7th, 8th, or 9th grades. Social behavior patterns may change during adolescence as adolescents transition from early, to mid, and to late adolescence and, concomitantly go from focusing on their peers' perspective to developing and acting according to their own identity within the peer group. So, we wanted to capture the social behavior patterns of a specific age group, namely mid-adolescence (i.e., aged roughly between 14 to 16 years old; Salmela-Aro, 2011). The informed consent forms sent out to parents and legal guardians explained the goals of the research, guaranteed the anonymity and confidentiality of the data to be collected, and provided ways to contact the research team if needed. After the school collected the parental consent forms, students were informed about the study and were asked to assent to their own participation which would consist of filling in the self-report questionnaires made available by their teacher using an online secure link developed using Lime survey^®^. Only the students within the conveniently selected schools attending the 7^th^ through 9^th^ grades that had parental consent and assented to participate were recruited.

Participants were 350 students aged 11 to 18 years old (M = 13.40, SD = 1.13), of which 191 (54.6%) were female and 159 (45.4%) were male. They attended the 7th (*n* = 149, 42.6%), 8th (*n* = 89, 25.4%) or 9th (*n* = 112, 32%) school grades, and the majority had never been retained in the same school year before (*n* = 289, 82.6%). Also, most students had never had psychological counseling (*n* = 202, 57.7%). The mean ages of male and female adolescents participating in this sample was found to be similar [*t*_(348)_ = 1.66, *p* = 0.09]. Also, the proportion of male and female adolescents was found to be similar across school years [$\chi_{\left(2\right)}^{2}$ = 1.84, *p* = 0.39], history of grade retention [$\chi_{\left(1\right)}^{2}$ = 3.61, *p* =0.06], and history of psychological counseling [$\chi_{\left(1\right)}^{2}$ = 3.20, *p* = 0.07].

### 2.2 Instruments

#### 2.2.1 Revised peer experience questionnaire (RPEQ)

The R-PEQ is a 28-item self-report scale that assesses the frequency with which overt (e.g., “*I chased a teen like I was trying to hurt him or her”*), relational (e.g., “*I left another teen out of an activity or conversation that they wanted to be included in”*), and reputational (e.g., “*I tried to damage another teens' social reputation by spreading rumors about them”*) aggressive and prosocial behaviors (e.g., “*I helped another teen when they were having a problem”*) are practiced and received. All items are answered twice, once about the individual practicing those acts toward others (i.e., the bully version as exemplified above) and another concerning the individual receiving those behaviors (i.e., the victim version not used in the current work; see below). Items are considered using a five-point scale from 0 (never) to 5 (several times a week), referring to the adolescent's experience during the last year. A four-factor measurement model for each version of the instrument was previously confirmed, and good indicators of internal consistency were found (α between 0.68 and 0.83 for the bully version and α between 0.78 and 0.84 for the victim version; Prinstein et al., 2001). Evidence has favored the same factor structure for the Portuguese version of the instrument that was used in the current work, and proof of construct validity in relation to another measure of aggressive behavior, psychopathic traits, and attachment to peers and parents was found. At least adequate internal consistency values also were found (α between 0.75 and 0.91 for the bully version and α between 0.76 and 0.88 for the victim version; Queirós and Vagos, 2016). Given the goals of the current work, only the bully measures of the RPEQ were used, and all achieved at least acceptable internal consistency values: α =0.79 for practicing overt aggression, α = 0.63 for practicing relational aggression, α = 0.81 for practicing reputational aggression, and α = 0.82 for practicing prosocial behavior. Though the relational aggression measure had a lower internal consistency value, it was still considered acceptable considering the goals of the current work, which focused on research purposes and on analyzing associations between variables and comparisons between groups, rather than classifying or making decisions on any given participant (Nunnally and Bernstein, 1994). Therefore, that measure was still included in the analysis.

#### 2.2.2 Mental health continuum-short form – for youth (MHC-SF)

The MHC-SF - for Youth is a 14-item self-report scale that assesses the degree of wellbeing based on diverse dimensions (i.e., emotional, social, and psychological) and previous evidence has widely favored its internal structure organized within those three measures (Iasiello et al., 2022). Reporting to the last month, youths are asked how often they felt what is described in each item, from never to every day. Three items assess emotional wellbeing (e.g., “*Happiness*”), five items assess social wellbeing (e.g., “*That you belong to a community*”), and the remaining six assess psychological wellbeing (e.g., “*Confident to think or express your ideas and opinions*”). The original version of the instrument obtained good internal consistency for the full scale (α = 0.74) and between acceptable and good internal consistency for the sub-scales (α = 0.59 and 0.70; Keyes, 2007). The Portuguese version, which was used in the current work, obtained good internal consistency values (α = 0.90 for the full scale and between 0.80 and 0.85 for the subscales; Matos et al., 2010). Using the current sample, internal consistency values were at least good for all measures: α = 0.81 for emotional wellbeing, α = 0.85 for social wellbeing, and α = 0.80 for psychological wellbeing.

### 2.3 Data analysis procedures

Data analyses were conducted using MPlus 7.4 (Muthén and Muthén, 1998–2017). Latent profile analyses were performed using the four RPEQ measures as indicators, namely the practice of overt, relational, and reputational aggression and prosocial behaviors. A one-profile model was firstly tested and then one profile was added at a time until no further improvement to the model fit indicators was found. The following fit indicators were considered for judging the fit of the models: (1) lower values of the information criteria, namely the Aikaike Information Criteria (AIC), the Bayesian Information Criteria (BIC), and the Sample Size Adjusted Bayesian Information Criteria (SSA-BIC) as indicating the best trade-off between model parsimony and residuals (Nylund et al., 2007); (2) entropy values higher than 0.70 referring to clearer proliferation and greater power to predict profile membership (Muthén, 2001); (3) Lo-Mendell Rubin and Bootstrap Likelihood Ratio Test *p*-values < 0.05 showing that k profiles are sufficient and k+1 profiles are likely not required (Nylund et al., 2007); and (4) Average probability of profile membership higher than 0.80 signifying a good profile solution (Muthén and Muthén, 2000). The sample size of the smallest profile was also carefully considered. If lower than 1% of the complete sample (*n* = 3) and/or *n* = 25, that profile should only be kept if it is theoretically sustained and informed (Muthén and Muthén, 2000). Having determined the optimal number of profiles, the mean differences between profiles on indicator variables (i.e., aggressive and prosocial behavior) was tested using the Wald pairwise chi-square tests (Arch, 2021) and on outcome variables relating to wellbeing using the BCH procedure (Asparouhov and Muthen, 2021). The probability of male and female participants being allocated to each profile was also tested using the DCAT procedure (Lanza et al., 2013).

## 3 Results

Table 1 shows the fit outcomes for the latent profile analyses. Based on the entropy, LMR/BLRT p-values, average probability values (i.e., average latent profile probability for profile 1 = 0.99 and for profile 2 = 0.95), and sample size for the smallest profile, a two-profile solution was considered the best fit (see Table 1).

**Table 1:** Model fit indicators for latent class analyses.

**Model**	**Log-likelihood**	**N.°of free parameters**	**AIC**	**BIC**	**SSA-BIC**	**Entropy**	**BLTR *p*-value**	**LMR *p*-value**	**Size of the smallest class**
1 class	−3362.48	8	6740.97	6771.83	6746.45	–	–	–	350
2 classes	−3116.34	13	6258.48	6308.63	6267.39	0.97	< 0.001	0.04	39
3 classes	−3046.75	18	6129.50	9198.94	6141.84	0.97	< 0.001	0.21	9

AIC, Akaike Information Criteria; BIC, Bayesian Information Criteria; SSA-BIC, Sample size adjusted Bayesian Information Criteria; BLRT *p*-value, *p*-value of the Bootstrap Likelihood Ratio Test, LMR, *p*-value of the Lo-Mendell-Rubin test.

**Figure 1:**
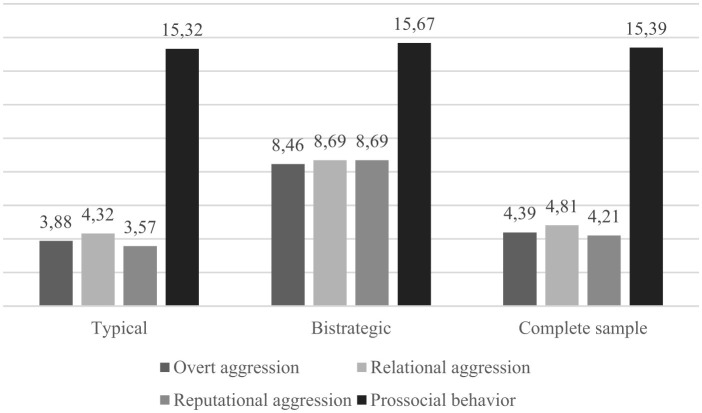
Mean scores for each class and the complete sample on aggression and prosocial behavior measures.

Descriptive values found for aggressive and prosocial behavior reported by participants in each profile are displayed in Table 2. Figure 1 shows a graphic representation of mean values of aggressive and prosocial behaviors reported by these profiles and by the complete sample. Profiles differed significantly in their reported practice of all forms of aggression but not in the practice of prosocial behavior. In relation to previously found mean values for a community Portuguese adolescent sample (i.e., M_overtaggression_ = 4.18, M_relationalaggression_ = 4.45, M_reputationalaggression_ = 3.86, M_prosocialbehavior_ = 15.22; Queirós and Vagos, 2016), profile 1 presented mean values close to those sample for aggressive and prosocial behavior, whereas profile 2 presented higher mean values for aggressive behavior and close to mean values for prosocial behavior. So, profile 1 (n = 311, 88.9%) was labeled “Typical,” and profile 2 (*n* = 39, 11.1%) was labeled “Bistrategic.”

**Table 2:** Between-class comparisons on aggression, prosocial behavior, wellbeing, and distribution by sex.

	**Typical**	**Bistrategic**	**Comparisons between profiles**
**Aggressive behavior**			
Overt aggression	3.86 (0.11)	8.44 (0.59)	*W =* −4.56, *p* < 0.001
Relational aggression	4.33 (0.09)	8.69 (0.66)	*W =* −4.36, *p* < 0.001
Reputational aggression	3.58 (0.09)	9.31 (0.64)	*W =* −5.73, *p* < 0.001
Prosocial behavior	15.31 (0.26)	16.05 (0.75)	*W =* −0.73, *p* = 0.36
**Wellbeing**			
Emotional	20.98 (0.34)	20.52 (1.14)	$\chi_{\left(1\right)}^{2}$ = 0.15, *p* = 0.70
Social	17.08 (0.41)	18.31 (1.12)	$\chi_{\left(1\right)}^{2}$ = 1.04, *p* = 0.31
Psychological	15.83 (0.32)	16.01 (0.88)	$\chi_{\left(1\right)}^{2}$ = 0.04, *p* = 0.85
**Sex**			
Female	0.57 (0.03)	0.32 (0.08)	$\chi_{\left(1\right)}^{2}$ = 9.19, *p* = 0.002
Male	0.43 (0.03)	0.68 (0.08)	

Values are presented as M (SE).

No significant differences between profiles were found for emotional, social, or psychological wellbeing. Finally, male and female adolescents had a similar probability of being allocated to the “Typical” profile. In contrast, boys were likelier to be assigned to the “Bistrategic” profile than female adolescents.

## 4 Discussion

Aggression and prosociality have mostly been addressed as incompatible social behaviors that are negatively correlated; likewise, they have individually been found to associate differently with wellbeing (e.g., Arslan et al., 2021; Son and Padilla-Walker, 2019) and to be diversely prevalent by gender (Queirós and Vagos, 2016). Alternatively, the RCT proposed that these behaviors may be used concomitantly as strategic ways to achieve diverse intra and interpersonal goals, namely social and individual status that may be particularly relevant to adolescent development. About this co-occurrence, previous works have categorized adolescents into a bistrategic group (Hawley, 2003; Wurster and Xie, 2014), but limited or inconsistent evidence has been found about how adolescents may be organized into different social profiles (Berger et al., 2015; Nantel-Vivier et al., 2014), particularly considering diverse forms of aggression. Also, how these different profiles may diverge in terms of subjective wellbeing has not been considered. Hence, the present study conducted latent profile analyses on measures of forms of aggression (overt, relational, and reputational) and prosocial behavior to explore their (co-)occurrence and then compare participant distribution by sex and self-reported wellbeing among different profiles. Using a person-centered approach through a latent profile analysis enabled the identification of meaningful subgroups within an adolescent sample that aligned with methodological procedures in a contemporary developmental and social psychology perspective (van der Gaag, 2023), considering that different behaviors may co-occur within individuals.

The best fit for the current sample of adolescents was a two-profile solution, depicting “Typical” adolescents who are within the expected values for both aggression and prosocial behavior in comparison with the complete sample and another community sample collected by Queirós and Vagos (2016), and “Bistrategic” adolescents who, comparably, resort more to aggression and similarly to prosocial behavior. Contrary to previous works that looked for profiles using peer-nomination to collect data (e.g., Berger et al., 2015; Hartl et al., 2019; McDonald et al., 2015), no evidence for mainly aggressive^1^ or mainly prosocial groups was found. Previous works had not used data collected via self-report questionnaires. Though such data collection strategy may be influenced by social desirability (Vigil-Colet et al., 2012), particularly in comparison with peer nomination strategies, it may also be the case that self- and other-perspective on ones' social behavior do not always coincide. So, the current work contributes to existing literature by pointing out the need to reflect these potential discrepancies, particularly considering that one's social behavior is likely driven by intrapersonal processes. The Social Information Processing theory has proposed such intrapersonal factors, which have been validated by previous systematic reviews (e.g., Martinelli et al., 2018) and empirical findings in relation to both aggressive (Vagos et al., 2025) and prosocial behavior (Laible et al., 2014b).

Most of the present study's sample was considered “Typical”, in as much as their scores for both aggression and prosociality were like those found for previous community adolescent samples comparable to the current one (Queirós and Vagos, 2016). Previous works have interpreted their profiles based solely on within-sample comparisons between participants. This group may resemble those adolescents Berger et al. (2015) classified as “normative non-aggressive” or who Hawley (2003), Wurster and Xie (2014) and Hartl et al. (2019) categorized as “Typical”: those adolescents who fell in no extreme in relation to the practice of both aggressive and prosocial behaviors and that also comprised most of those works' samples. Regarding gender distribution, our findings align with those of Wurster and Xie (2014) and of McDonald et al. (2015), who found a similar distribution of male and female adolescents in the “Typical” group.

Unlike previous works that have used latent profile analyses to explored how adolescents might be distributed based on profiles or groups (Berger et al., 2015; Hartl et al., 2019), this study found evidence for a “bistrategic” group of adolescents who resort more to aggression (significantly more than the “typical” group and descriptively more than mean values of a community comparable sample; Queirós and Vagos, 2016) but also resort to prosocial behavior at least as much as can be expected (i.e., similar to the “Typical group” and to another community comparable sample). These findings are more aligned with a developmental perspective on adolescence that has found evidence of adolescents who continue to resort to both prosocial and aggressive behavior over time (Ettekal and Mohammadi, 2020; Nantel-Vivier et al., 2014). Though we intended to capture a specific age group based on school year, our sample has a wide age range (i.e., 11 to 18 YO) and that age-diversity may have been able to grasp that developmental pathway. In this case, both behaviors may co-exist and be used by adolescents to fulfill social functions and adapt to society, namely relating to one's social status within the group, in accordance with the RCT (Hawley, 1999). In fact, prosocial behavior may be seen as a reparatory process (i.e., to reverse or make amends) that bistrategic adolescents employ after having reacted aggressively (Arbel et al., 2022).

Adolescents in the “bistrategic” profile reported similar levels of all forms of aggression, alike previous findings by Hartl et al. (2019), meaning that indirect aggression does not particularly coadunate with prosociality and its associated social gains. Based on the present study findings, it would seem that bistrategic adolescents may not only resort to different structural behaviors (i.e., aggressive and prosocial behavior) in relation to individual and social goals but also to diverse forms of the same structural behavior, namely the various forms of aggression; the co-occurrence of the diverse forms of aggression was to be expected based on previous literature (Ingram, 2014). It may be the case that profiles are distinct based primarily on the intention behind the act rather than the frequency of the act itself: the combined intention may be to obtain gains or increase ones' social status, either by damaging a victim directly or indirectly or by leading others to a more socially acceptable view of the self. Pfattcheicher et al. (2022) discuss the intentionality of prosocial behavior as not necessarily being directed at others' welfare and rather distinguish prosociality from altruism based on that intention. Likewise, McGinley and Carlo (2006) proposed that prosocial behavior should be seen as a complex construct that includes different types of behaviors (e.g., altruism, public prosocial behaviors, compliant prosocial behaviors, dire, emotional, and anonymous prosocial behaviors) and that some of these behaviors could actually be used to perpetrate or potentiate aggressive intentions, mainly on its reputational and relational forms. Intentionality and values have also been proposed to distinguish adolescents social behavior. Findley and Ojanen (2013) used intentionality (i.e., dominate resources based on social actions) to categorize adolescents into groups, including bistrategic adolescents, and found them to score higher on both physical (i.e., direct) and reputational (i.e., indirect) aggression compared to mainly prosocial, mainly aggressor or typical adolescents. Finally, McDonald et al. (2015) found that bistrategic adolescents valued contributing to others welfare as much as maintaining the social hierarchy and status quo of their peer groups, in comparison with aggressors who favored achieving power.

Unlike previous works (e.g., McDonald et al., 2015; Hartl et al., 2019), current findings indicate that male adolescents were more prevalent than female adolescents in this bistrategic group. This being an European sample – unlike those works that used a North American and Chile sample—it may be that males' higher practice of all forms of aggression (Vagos et al., 2014) that is found for European but not American samples (Voulgaridou and Kokkinos, 2023) has made male participants more visible within this group characterized by such acts.

Both profiles showed similar levels of wellbeing, in all its dimensions. In other words, adolescents experience positive emotions (i.e., emotional wellbeing), a sense of personal development and purpose (i.e., psychological wellbeing), and a sense of being a relevant part of a community (i.e., social wellbeing) regardless of their differences in aggressive behavior. This finding may be driven by the same levels of prosocial behavior reported by both profiles, in as much as previous works have consistently shown that prosocial behavior is a facilitator of mental health over time, even in the presence of aggression. Specifically, prosocial behavior has been found to be protective against emotional problems (Memmott-Elison and Toseeb, 2023) and problematic behaviors (Memmott-Elison and Toseeb, 2023; Williams et al., 2024) from infancy to adolescence (Memmott-Elison and Toseeb, 2023) and within adolescent years (Padilla-Walker et al., 2018), and to promote peer acceptance of children who are both prosocial and aggressive (Ettekal and Mohammadi, 2020). Considering bistrategic adolescents in particular, those who resort to both social and coercive intentions are also more liked and more popular than most other groups, but particularly in comparison with the mainly aggressor group (Findley and Ojanen, 2013). Current findings add to previous ones by highlighting the intrapersonal gains of being bistrategic, in addition to the social gains that had been the focus of previous research.

## 5 Implications for applied settings

The present study's findings reinforce that social behaviors are used as a strategic way to navigate the complexities of the social world during adolescence (Findley and Ojanen, 2013) and echo previous works asserting that promoting prosocial behavior be proposed as a relevant intervention to continuously mitigate the negative outcomes associated with aggression (Laible et al., 2014a; Williams et al., 2024). We would further propose that prosocial behavior be promoted in its diverse forms and to be aligned to diverse individual and social goals; in other words, prosocial behavior does not need to be seen solely as putting others' welfare in front of ones' own (i.e., altruism; Pfattcheicher et al., 2022) but rather as a way to demonstrate willingness to promote intra and interpersonal gains. The relevance of considering the intentionality of the act, in addition to its manifest behavior and potentially associated costs and benefits, seems particularly relevant as some evidence exists that prosocial behaviors may be practiced in response to anxiety, particularly toward friends in adolescence (Padilla-Walker et al., 2015), or to be seen by an audience and in this case to be negatively associated with diverse dimensions of wellbeing, life purpose, relationship with others or self-acceptance (González-González and Betancourt-Ocampo, 2021). Considering these aspects could add to the promising evidence that promoting prosocial behavior has more effect than (or in combination with) preventing problem behaviors (Shin and Lee, 2021).

In addition to interventions focused on the individuals to promote prosocial behaviors, professionals in schools could also benefit from training to recognize that prosocial behavior may coexist with aggression, and that the promotion of prosociality without addressing underlying motives might not be sufficient, highlighting the need for tailoring programs to address the function and intention behind behaviors, rather than just categorizing them. The need for initial and continued professional development of teachers in relation to recognizing and understanding bullying has been proposed as an important asset to mitigate the growing experience of bullying and aggression within schools (O'Brien et al., 2023).

## 6 Limitations

Firstly, this study relied only on self-reported measures that, despite their previous psychometric appraisal, could be susceptible to bias, namely social desirability. Furthermore, as a cross-sectional study, there are limits to the understandability of the associations between variables. Previous works have found that social behaviors differ throughout different stages of development (Wright and Wachs, 2019). These changes may be driven by or bring along changes in wellbeing, something that could only be ascertained using a longitudinal design. Finally, specific features of the currents' work sample should be considered carefully when interpreting and generalizing findings, namely the samples' size, age-range and cultural specificities. Though sample size surpasses the necessary to achieve statistical power while also being similar to that used previously (e.g., Findley and Ojanen, 2013; Wurster and Xie, 2014), it is smaller than samples where profiles were empirically tested (e.g., Berger et al., 2015; Padilla-Walker et al., 2018) and may have resulted in some relevant profiles (e.g., mainly aggressors or mainly prosocial adolescents) being missed by our analyses. Though our samples' age range is large, most participants are within the mid-adolescence age range (i.e., M = 13.40, SD = 1.13). Mid adolescents are particularly influenced by peers, compared to early and late adolescents, towards whom adolescents experience their newly found individual identity and try to fit in. Again, the focus in developing intimate and supportive relationships within this developmental life period may have sustained that both profiles found in the current work are attuned to achieving social gains. Current findings may also reflect a specific cultural conservative context where individuals strive to avoid risk and the unknown (i.e., high uncertainty avoidance) and prefer to live collectively (i.e., low individualism), unlike the cultural specificity of, for example, the North-American society (Hofstede Insights, 2023, Country comparison tool). This more collective and risk avoidance characteristics may sustain that the current work found profiles mostly driven by sustaining social gains, by using mostly prosocial behavior or by strategically combining it with aggressive behavior.

## 7 Conclusions

Integration into the peer group is critical during adolescence and depends on the social behavior practiced by the adolescent, including aggressive and prosocial behaviors. Though these behaviors have often been treated as seemingly opposing constructs, it has been theoretically proposed (Hawley, 1999; Hawley and Bower, 2018) and empirically validated (e.g., Wurster and Xie, 2014) that being able to use both kinds of behaviors may have strategic value in attaining intra and interpersonal resources. Findings from the present study partially align with the RCT model, in as much as results showed two profiles that differentiate in the practice of aggression but not in the practice of prosocial behavior and that were, hence, labeled typical or bistrategic adolescents. Such findings highlight the importance of considering self-reported perspectives on ones' social behavior when considering social behavior profiles, as well as understanding how those profiles relate to intrapersonal variables, specifically wellbeing. It seems that bistrategic individuals have both social and individual gains, even if they are experiencing the costs of also being involved in conflicting relationships. The intervention challenge will be to disentangle aggressive and prosocial behaviors, which may be better achieved if resorting to both individual (e.g., addressing ones' values or goals in relation to social interactions) and contextual (e.g., addressing norms defining how to achieve popularity) changes.

## Data Availability

The datasets presented in this article are not readily available. The raw data supporting the conclusions of this article will be made available by the authors, upon reasonable request. Requests to access the datasets should be directed to leniac@upt.pt.

## Footnotes

^1^A three-profile solution showed this group that had only 9 participants. This three-profile solution was not considered the best fit for our data.
